# Development and assessment of diabetic nephropathy prediction model using hub genes identified by weighted correlation network analysis

**DOI:** 10.18632/aging.204340

**Published:** 2022-10-14

**Authors:** Xuelian Zhang, Yao Wang, Zhaojun Yang, Xiaoping Chen, Jinping Zhang, Xin Wang, Xian Jin, Lili Wu, Xiaoyan Xing, Wenying Yang, Bo Zhang

**Affiliations:** 1 Department of Endocrinology, China-Japan Friendship Hospital, Beijing 100029, People’s Republic of China

**Keywords:** diabetic nephropathy, prediction model, risk factors, weighted correlation network analysis

## Abstract

Diabetic nephropathy (DN) is one microvascular complication of diabetes. About 30% of diabetic patients can develop DN, which is closely related to the high incidence and mortality of heart diseases, and then develop end-stage renal diseases. Therefore, early detection and screening of high-risk patients with DN is important. Herein, we explored the differences of serum transcriptomics between DN and non-DN in type II diabetes mellitus (T2DM) patients. We obtained 110 target genes using weighted correlation network analysis. Gene Ontology enrichment analysis indicates these target genes are mainly related to membrane adhesion, alpha-amino acid biosynthesis, metabolism, and binding, terminus, inhibitory synapse, clathrinid-sculpted vesicle, kinase activity, hormone binding, receptor activity, and transporter activity. Kyoto Encyclopedia of Genes and Genomes analysis indicates the process of DN in diabetic patients can involve synaptic vesicle cycle, cysteine and methionine metabolism, N-Glycan biosynthesis, osteoclast differentiation, and cAMP signaling pathway. Next, we detected the expression levels of hub genes in a retrospective cohort. Then, we developed a risk score tool included in the prediction model for early DN in T2DM patients. The prediction model was well applied into clinical practice, as confirmed by internal validation and several other methods. A novel DN risk model with relatively high prediction accuracy was established based on clinical characteristics and hub genes of serum detection. The estimated risk score can help clinicians develop individualized intervention programs for DN in T2DM. External validation data are required before individualized intervention measures.

## INTRODUCTION

Type 2 diabetes mellitus (T2DM) is chronic metabolic disease caused by defective insulin secretion and/or insulin resistance [1]. The stimulation of chronic hyperglycemia activates various intracellular signal transduction pathways, and produces glucose toxic products, which in turn damage capillary endothelial cells, resulting in capillary basement membrane thickening, extracellular matrix deposition, and finally systemic microvascular diseases [2, 3]. Type 2 diabetic nephropathy (T2DN) is one of its main microvascular complications [4, 5]. The pathological changes of early nephropathy mainly involve the glomeruli distributed in the renal cortex, and the glomeruli are composed of many capillary clusters [6]. The onset of T2DN is insidious. Once it enters the massive proteinuria stage, the progression rate to end-stage renal disease is about 14 times that of other kidney diseases [7, 8]. Therefore, early diagnosis, prevention and delay of the occurrence and development of DN can improve the survival rate of T2DM patients. Improving their quality of life is of great significance.

In the present study, we first identified the hub genes that may be involved in the process of DN using weighted correlation network analysis (WGCNA). Next, we developed DN risk prediction models based on target genes and clinical parameters. Then, we evaluated and validated the prediction ability of the models. Finally, we built a nomogram risk tool to evaluate the DN risk of individuals. Our study will help clinical decision-making and improve the prognosis of T2DM patients.

## MATERIALS AND METHODS

### GEO datasets

We downloaded datasets GSE142153 and GSE26168 (both expression profiling by array) from the GEO database (https://www.ncbi.nlm.nih.gov/gds). The GSE142153 based on the GPL6480 platform includes 3 condition experiments: Healthy control (n=10), DN (n=23), and end-stage renal disease (n=7). The tissue type was from peripheral blood. The GSE26168 based on the GPL6883 platform includes healthy controls (n=8), impaired fasting glucose group (n=7), and T2DM group (n=9). We used the DN group of GSE142153 and the T2DM group of GSE26168 for further analyses.

### Patients

This is a retrospective cohort study design. The patients included here had T2DM in the hospital. We collected the baseline data from November 2013 to June 2014, and acquired the follow-data from September 2017 to June 2018. We identified the T2DM according to one or more of the following criteria: HbA1c ≥ 6.5%, fasting blood glucose ≥ 7.0 mmol/L, oral glucose tolerance test ≥ 11.1 mmol/L, and random blood glucose ≥ 11.1 mmol/L [9]. The criteria for inclusion were: age under 75 years old; no severe disease including malignant tumor, uremia dialysis, chronic inflammatory disease, autoimmune diseases, and cerebra-cardiovascular disease. Patients are conscious and can communicate with the investigator. Patients with >30 mg/g (albumin /creatinine ratio) at baseline, or incomplete data or information were excluded. This study was approved by the Ethics Committee of China-Japan Friendship Hospital, and all subjects provided written informed consent.

### Bioinformatics analysis

With R packages “limma” and “sva”, we first merged the DN group of GSE142153 and the T2DM group of GSE26168. We used the “removeBatchEffect” function in the R package limma. Differential expression analyses were performed between the two groups. The differentially expressed genes (DEGs) were identified using the following criteria: |log2 fold change|>2 and adjusted P <0.05.

WGCNA is a system biology method used to describe gene association patterns between different samples [10, 11]. It can be used to identify highly covarying gene sets and to find out candidate biomarker genes or therapeutic targets based on the interconnectedness of gene sets and the association between gene sets and phenotypes. The WGCNA consists of gene co-expression network construction, and identification of modules, related modules to external information, relationships between modules, and key drives in interesting modules. We used the following criteria to identify the related modules: the module size of 10-20000, the cut height equal to 0.25 and verbose equal to 5. Highly related genes were identified with thresholds greater than 0.1 in the topological overlap matrix (TOM).

We made an overlap between DEGs and related module genes identified by WGCNA. The intersection genes were used for further analyses. Gene Ontology (GO) annotation and Kyoto Encyclopedia of Genes and Genome (KEGG) pathway analyses were achieved using R packages “clusterProfiler”, “enrichplot” and “ggplot2”, and the results were presented in bar plots [12, 13]. We further established protein-protein interaction (PPI) using String (https://www.string-db.org/). Any gene without nodes with other genes was excluded. The PPI was inputted into Cytoscape [14]. Using the application of cytoHubba, we identified the top 10 hub genes using five algorithms: maximal clique centrality (MCC), maximum neighborhood (MN), maximum neighborhood component (MNC), Degree, and edge percolated component (EPC). Intersections among the five algorithms were made, which were considered as the key genes.

### Clinical data collection

The following information was collected for each patient: age, gender, smoking status, drinking status, body mass index (BMI, weight/height^2^, kg/m^2^), systolic blood pressure, and diastolic blood pressure. The venous blood was extracted before receiving any treatment. The fasting blood glucose (FBG), HbA1c, total cholesterol (TC), triglyceride (TG), high-density lipoprotein (HDL), low-density lipoprotein (LDL), blood urea nitrogen (BUN), and serum creatinine (SCr) were measured using the Jaffe rate-blanked compensated creatinine assay. Urine acid (UA) was detected using an automated biochemical analyzer. The normal and abnormal standards were from the Guidelines for Prevention and Treatment of Dyslipidemia in Chinese Adults [15]. Patients with urinary albumin creatinine ratio >30 mg/g were diagnosed with DN [16]. According the World Health Organization, people who have smoked continuously or cumulatively for 6 months or more were defined as smokers. Men and women who drank more than 60 g and 40 g of pure alcohol per day respectively were considered as drinking. BMI was calculated as BMI = weight (kg)/height (m)^2^. Hypertension was defined as systolic blood pressure ≥ 140 mmHg and/or diastolic blood pressure ≥ 90 mmHg.

For evaluating the expression levels of seven hub genes, we extracted the total RNA from the peripheral blood of each T2DM patient using a modified RiboPure™-Blood kit (Ambion, Austin, USA) according to the manufacturer’s protocol. The cDNA was synthesized using a reverse transcription kit (Guangzhou Ribobio, Co., Ltd., China). Quantitative real-time polymerase chain reaction (qRT-PCR) was carried out using a Cobas Z480PCR system. The relative expressions of the hub genes were calculated using the 2^-ΔΔ^*^CT^* method. The forward and reverse primer sequences were as follows: TTCTCAGAGGCTGACATGCGCT and CTCGTCCAGAAGGATGTTGGCT (ADRBK1); CATCGAGCAGAGTGTCTACAGC and TGTCGTCGCTATCCAGG TCATG (LMX1A); CACCACGCTCTCCAATGCCTTT and CATCACCAGGATGG ACACAAGC (HTR1B); GACAACCACTGTGAACATCACGC and TGACACTTC CAATGGCTGTGCC (CDH10); TTACTGGTCCGACTGGTACGAC and CAAAGA ACTGCTGAGGCTTGGG (TAC1); CTCCTCTTTGTCATCACGCTTCC and GGA TGAGGACACTGCTGTAGAG (NPY); CTGGAACGTGACCAACGCCATC and TCATTCTCCTCGTACAGGCACG (SLC32A1).

### Statistical analysis

All continuous variables were transformed into categorical variables, and expressed using counts and percents like other original categorical variables. We first screened relevant genes from the seven hub genes using the LASSO method, and then calculated the risk score for each patient as follows: risk score =gene_1_ expression*β_1_+…+gene_n_ expression*β_n_. Then the risk score was inputted as a continuous variable into logistic regression. We established three models for the prediction of DN: model 1 with only clinical parameters, model 2 with only identified gene expression, and model 3 with combined clinical parameters and gene expression. Multivariate logistic regression was used to validate if the risk score was an independent predictor of DN among T2DM patients or not. The receiver’s operating characteristic (ROC) curves were used to assess the discrimination ability of the models. Nomograms were adopted to quantify the predictive ability of the models. Calibration curves were plotted to evaluate the calibration of DN risk, which was accompanied by a Hosmer-Lemeshow test. Decision curve analysis was conducted to evaluate the clinical practicability of the models based on the net benefit according to the different threshold probabilities among T2DM patients.

### Data availability

The expression profiling of genes from diabetics and diabetics nephropathy can be available from the GEO (GSE142153 and GSE26168). The other data can be obtained from the corresponding author (Bo Zhang).

## RESULTS

### Differential expression analysis of genes

**Figure 1:**
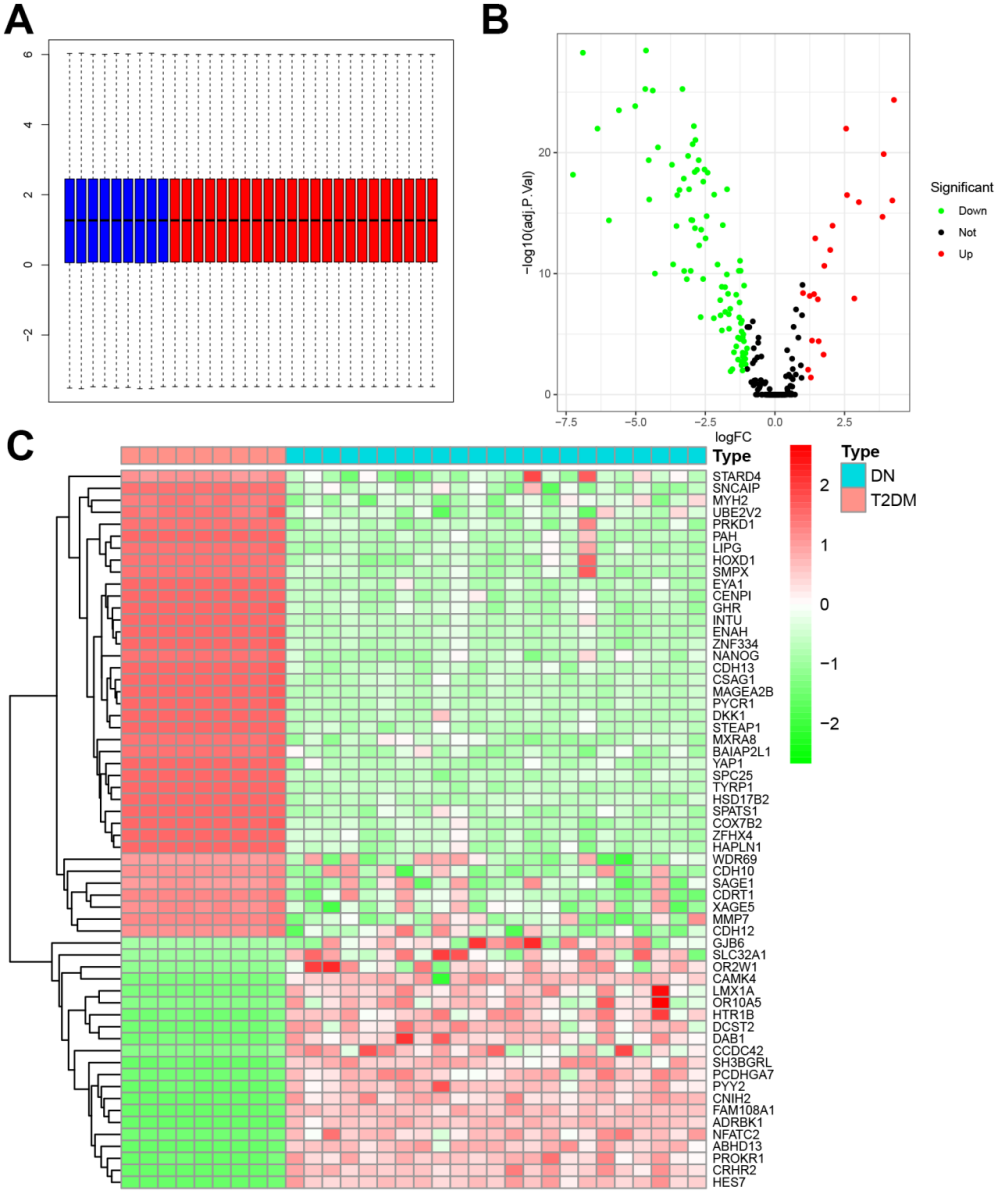
**Differentially expressed genes between GSE30529 and GSE30528.** (**A**) Normalized boxplot of control and DN groups. (**B**) Volcano plot of differentially expressed genes. (**C**) Heatmap of differentially expressed genes between control and DN groups.

Using 23 DN samples and 9 diabetic samples, we performed differential expression analysis to obtain the DEGs that can be associated with DN. First, we merged the expression data of the DN group of GSE142153 and the T2DM group of GSE26168 to exclude the bath effects. The boxplot showed the whole gene expression levels of the merged dataset were approximately the same, which indicated no batch effect (Figure 1A). Using the criteria of |log2-fold changes (FC)|>1.0 and adjusted P <0.05, we obtained 110 DEGs, including 9 upregulated DEGs and 101 downregulated DEGs between the two groups (Figure 1B). The top 60 DEGs were presented in the hierarchical clustering heatmap (Figure 1C), including the significantly upregulated or downregulated DEGs.

### Weighted gene co-expression network analysis (WGCNA)

**Figure 2:**
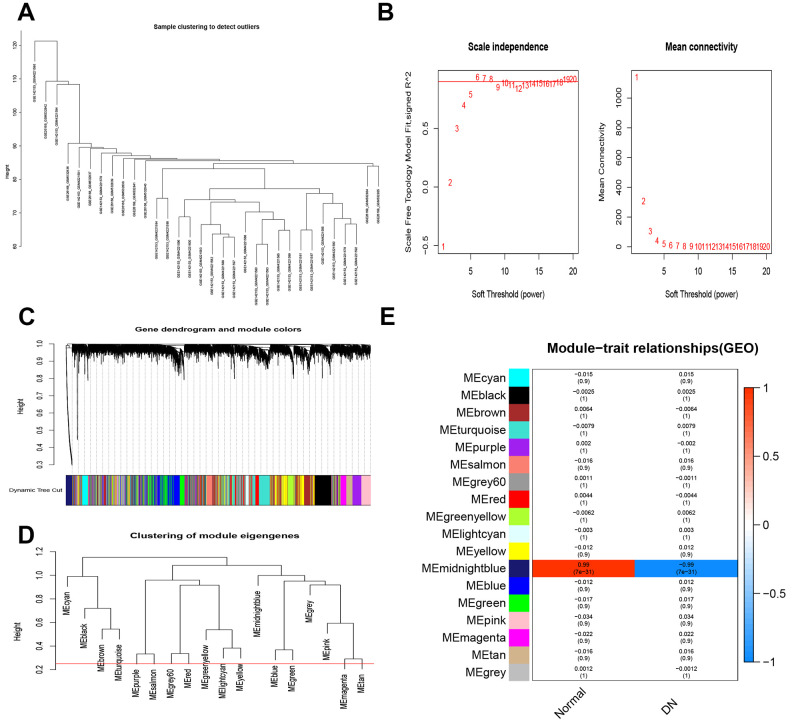
**Weighted gene co-expression network analysis.** (**A**) Sample clustering. (**B**) Analysis of soft-thresholding powers to fit the scale-free topology model and the mean connectivity of the soft-thresholding powers. (**C**) Dendrogram of the gene modules. (**D**) Clustering of 8 gene modules. (**E**) Module-trait relationships.

**Figure 3:**
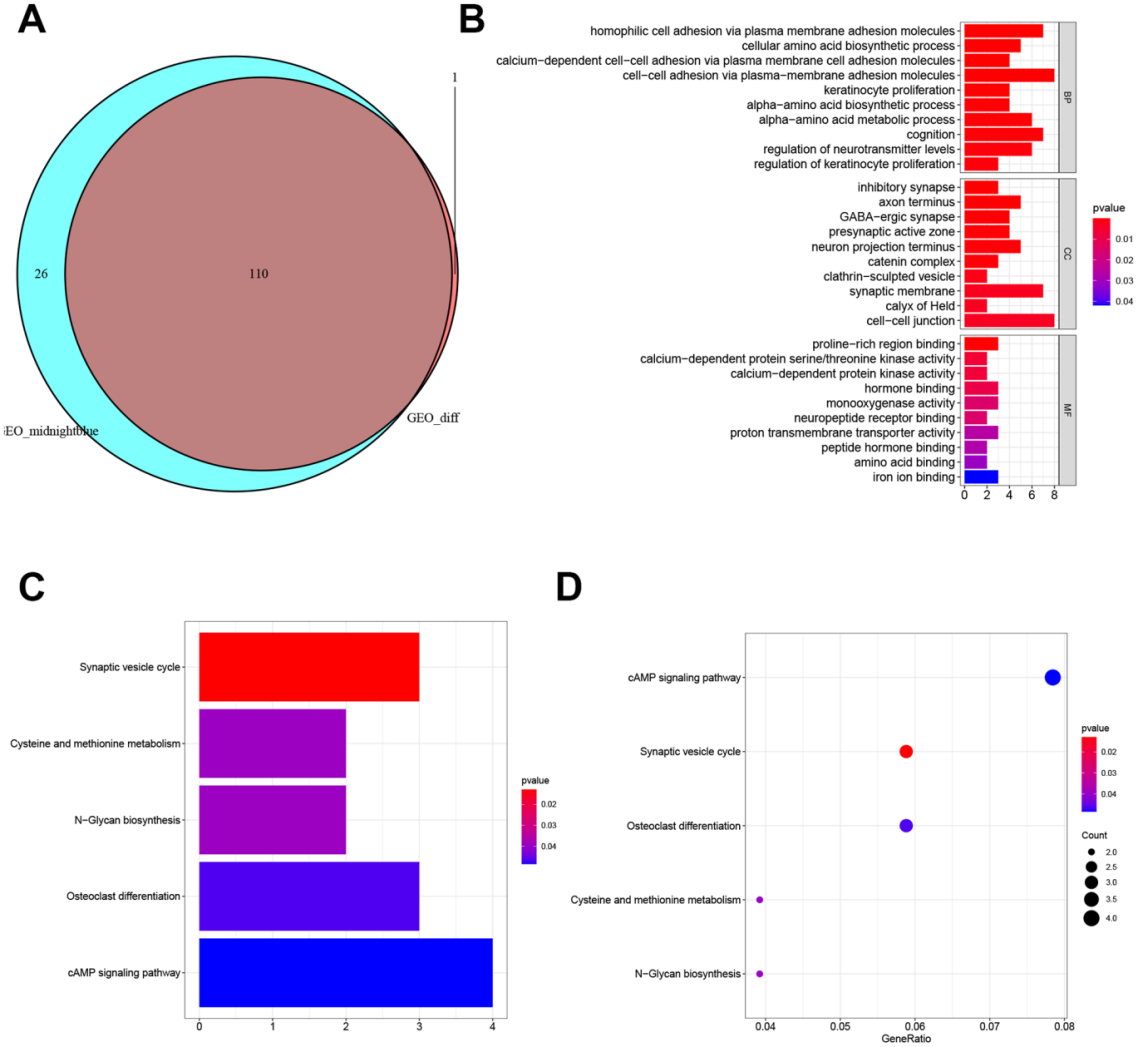
**Enrichment and pathways analysis.** (**A**) Venn diagrams of DEG list and highly-related gene list. (**B**) GO enrichment analysis of 130 gens. (**C**, **D**) KEGG pathway analysis of 130 genes.

Using WGCNA that describes the expression correlation among multiple genes, we identified the genes of interest. Two analytical methods were applied to identify the target genes. First, we performed the cluster analysis to assess sample similarity. The outlier’s detection identified three samples that were removed (GSM4221586, GSM532842, GSM4221594, Figure 2A). To construct a scale-free network, we performed WGCNA, and the scale-free topological fitting index was 0.88 and the mean connectivity was 90 by setting the soft threshold power at 10 (Figure 2B). Furthermore, we carried out hierarchical clustering to identify different kinds of gene modules using weighted gene co-expression correlation, which can be found through the branches of clustering tree and different colors. We identified 18 modules in size from 20 to 10000 in the network, and merged cut highs of 0.25 (Figure 2C). The 18 modules can be separated into three clusters according to the correlations among the modules (Figure 2D). Besides, the weighted coexpression correlations of all genes were presented in a heatmap plot (Figure 2E). The module MEmidnightblue showed the highest correlations, the correlation coefficients between the control group and the DN group were 0.99. Finally, we obtained 136 highly-related genes in the TOM matrix with a threshold greater than 0.1. The results of the two analyses can be overlapped, and a list of 110 target genes were identified, which may be involved in DN (Figure 3A).

### Function enrichment analysis of targeted genes

**Figure 4:**
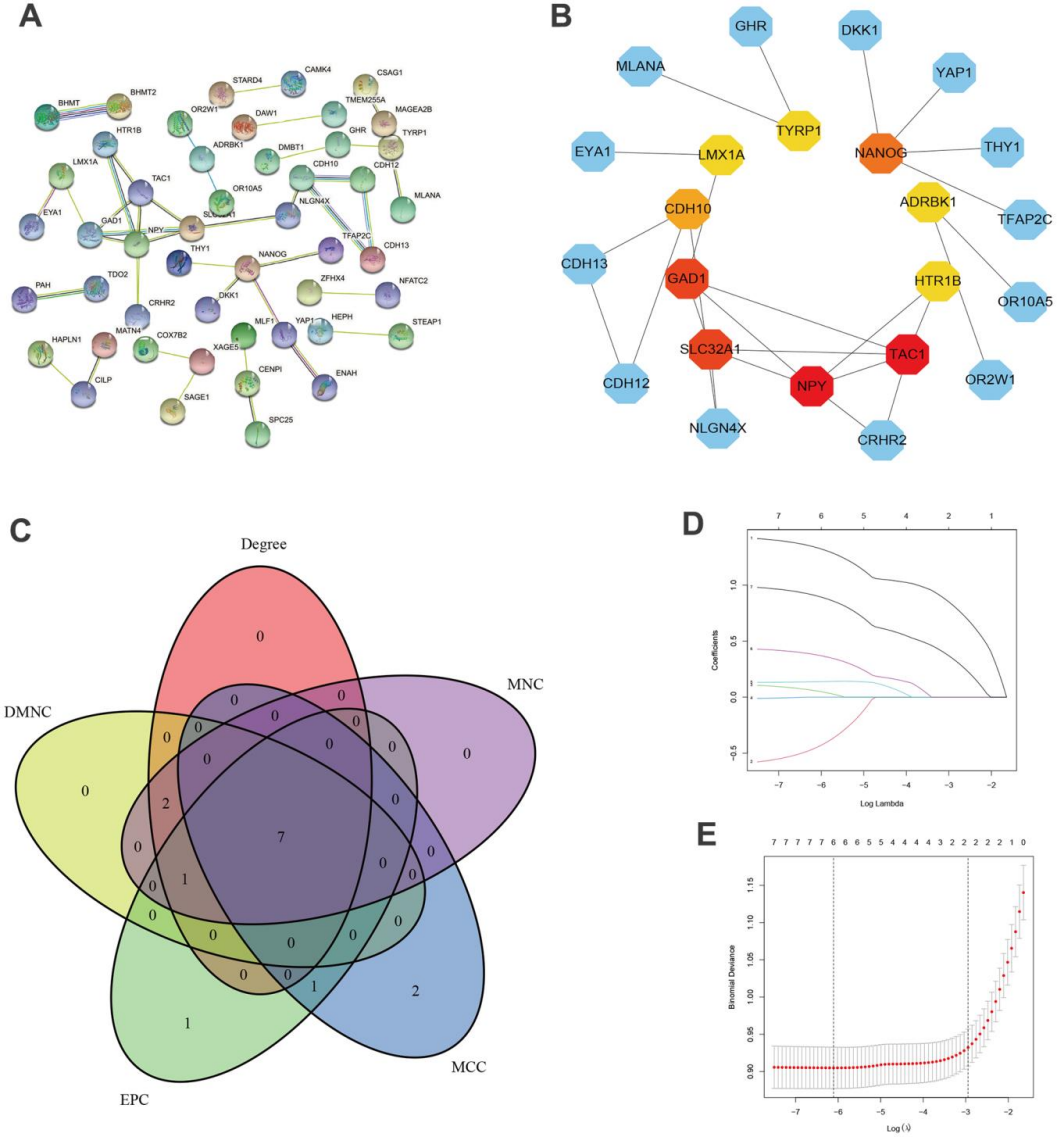
**PPI networks.** (**A**) PPI network of combined genes. (**B**) PPI network of top 10 Hub genes. (**C**) Venn diagrams of Hub genes based on MCC, MNC, Degree, DMNC, EPC methods. (**D**) Cross-validation for identifying parameters in LASSO. (**E**) LASSO regression of 6 hub genes.

The occurrence of DN may involve complex pathogenesis and biological processes, and understanding the potential function of target genes will help with DN treatment. The GO enrichment analysis indicates these target genes are mainly related to membrane adhesion, alpha-amino acid biosynthesis, metabolism, and binding, terminus, inhibitory synapse, clathrinid-sculpted vesicle, kinase activity, hormone binding, receptor activity, and transporter activity (Figure 3B). KEGG analysis indicates synaptic vesicle cycle, cysteine and methionine metabolism, N-Glycan biosynthesis, osteoclast differentiation, and cAMP signaling pathway can be involved in the process of DN in diabetic patients (Figure 3C, 3D). We also built the PPI based on these target genes (Figure 4A), and identified the top 10 hub genes: LMX1A, TYRP1, NANOG, ADRBK1, HTR1B, TAC1, NPY, SLC32A1, GAD1, and CDH10 (Figure 4B). Finally, we identified the hub genes using five algorithms (Figure 4C). We overlapped the results of the five algorithms and obtained the final hub genes that were used for model building: ADRBK1, LMX1A, HTR1B, CDH10, TAC1, NPY, and SLC32A1.

### Development of DN prediction model based on target genes

Based on 7 target genes, we performed LASSO regression and identified 6 genes (ADRBK1, LMX1A, HTR1B, TAC1, NPY, SLC32A1) included in the prediction model (Figure 4D). We calculated the risk score as follows: risk score = 1.466 * ADRBK1 - 0.627 * LMX1A + 0.199 * HTR1B + 0.130 * TAC1 + 0.477 * NPY + 1.005 * SLC32A1.

**Figure 5:**
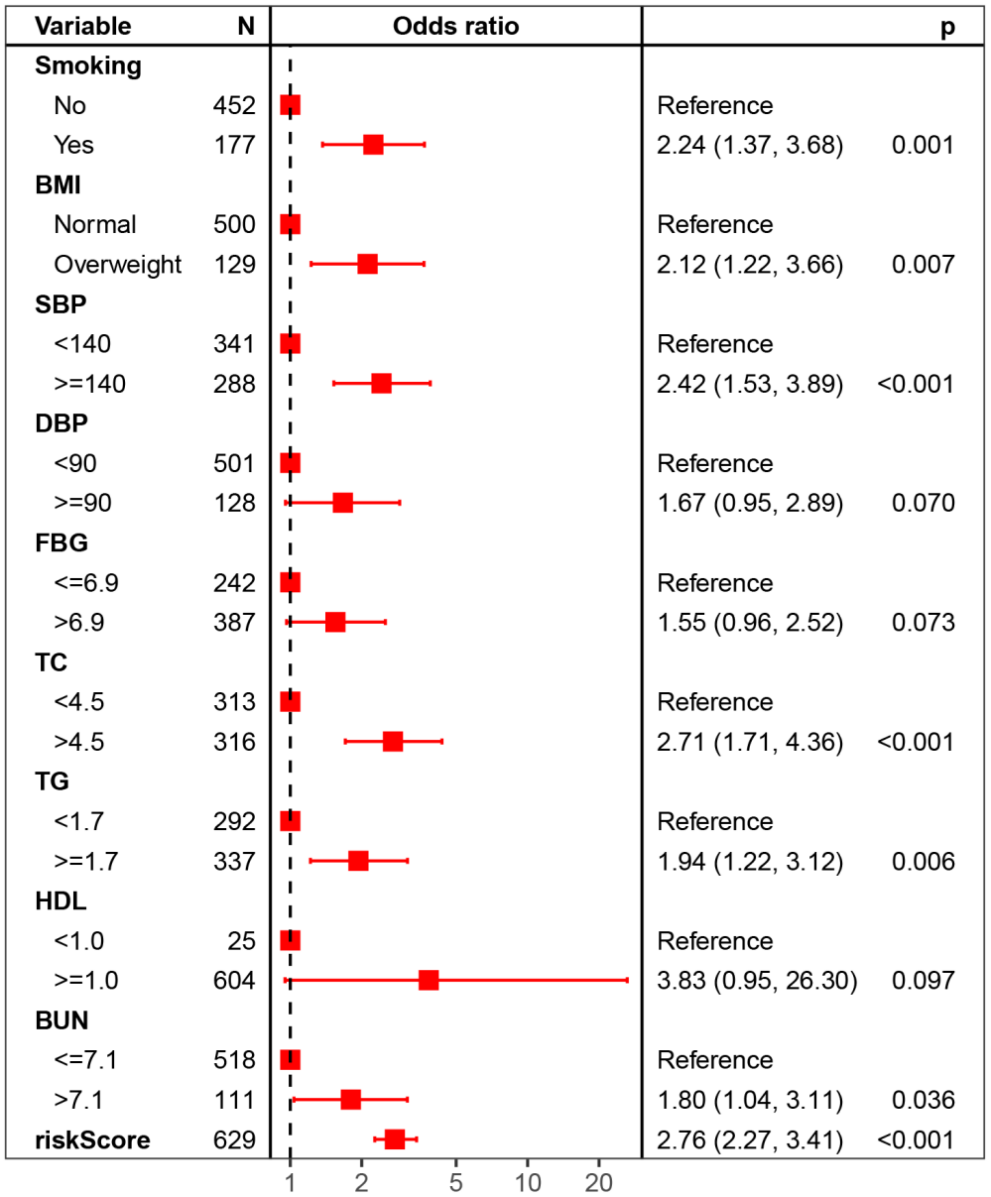
Forest plot of multivariate logistic regression for DN risk.

The general characteristics between the DN group and the non-DN group were compared. The risk scores were higher in the DN group than in the non-DN group (10.54 vs. 9.03, *P*<0.001; Table 1). The DN group had higher proportions of males (65.4% vs. 55.9%, *P*=0.042) and smokers (42.6% vs. 23.1%, *P*<0.001). The SBP (P<0.001) and FBG (P=0.016) levels were higher in the DN group than in the non-DN group. The TC, TG and BUN levels were also significantly elevated in the DN group (P<0.001). No significant differences were observed in age >60, drinking status, DBP>=90 rate, and HbA1c, HDL, LDL, SCR, or UC levels (*P*>0.05). The univariate logistic regression indicated the risk score was positively associated with DN (OR (95%CI): 2.72 (2.26-3.26), *P*<0.001). Besides, male (1.49 (1.03-2.17), P=0.034), FBG>6.9 (1.62 (1.11-2.38), P=0.013), overweight or obesity (1.84 (1.21-2.78), *P*=0.004), DBP>90 (1.55 (1.02-2.37), P=0.042), and smoking (2.47 (1.69-3.60)), SBP>140 (2.23 (1.55-3.22)), abnormal TC (2.68 (1.84-3.91)) and TG (2.16 (1.48-3.14)), and elevated BUN (2.45 (1.60-3.77)) (all P<0.001) were also risk factors of DN in diabetic patients (Table 2). Using these significant variables in the univariate logistic regression, we performed multiple logistic regression. Our results indicated that risk score was an independent risk factor of DN for diabetic patients (OR:2.76, 9%CI: 2.27-3.41, P<0.001). Besides, smoking, overweight, SBP>=140, abnormal TC, TG and BUN were also risk factors of DN (Figure 5).

**Table 1:** Comparisons of general characteristics between control and case group.

**Parameters**	**Level**	**Control, n (%)**	**Case, n (%)**	***P***
Age (years)	≤60	199 (42.6)	57 (35.2)	0.118
	>60	268 (57.4)	105 (64.8)	
Gender	Female	206 (44.1)	56 (34.6)	0.042
	Male	261 (55.9)	106 (65.4)	
Smoking	No	359 (76.9)	93 (57.4)	<0.001
	Yes	108 (23.1)	69 (42.6)	
Drinking	No	299 (64.0)	107 (66.0)	0.712
	Yes	168 (36.0)	55 (34.0)	
SBP (mmHg)	<140	277 (59.3)	64 (39.5)	<0.001
	≥140	190 (40.7)	98 (60.5)	
DBP (mmHg)	<90	381 (81.6)	120 (74.1)	0.053
	>=90	86 (18.4)	42 (25.9)	
FBG (mmol/L)	≤6.9	193 (41.3)	49 (30.2)	0.016
	>6.9	274 (58.7)	113 (69.8)	
HbA1c (%)	≤6.9%	230 (49.3)	71 (43.8)	0.272
	>6.9%	237 (50.7)	91 (56.2)	
TC (mmol/L)	≤4.5	261 (55.9)	52 (32.1)	<0.001
	>4.5	206 (44.1)	110 (67.9)	
TG (mmol/L)	<1.7	239 (51.2)	53 (32.7)	<0.001
	≥1.7	228 (48.8)	109 (67.3)	
HDL (mmol/L)	<1.0	23 (4.9)	2 (1.2)	0.066
	≥1.0	444 (95.1)	160 (98.8)	
LDL (mmol/L)	<1.8	307 (65.7)	108 (66.7)	0.906
	≥1.8	160 (34.3)	54 (33.3)	
BUN (mmol/L)	≤7.1	402 (86.1)	116 (71.6)	<0.001
	>7.1	65 (13.9)	46 (28.4)	
SCR (μmol/L)	≤106	436 (93.4)	148 (91.4)	0.499
	>106	31 (6.6)	14 (8.6)	
UC (mmol/L)	≤440	425 (91.2)	141 (87.0)	0.168
	>440	41 (8.8)	21 (13.0)	
Risk Score (mean (SD))		9.03 (1.18)	10.54 (1.22)	<0.001

**Table 2:** Results of univariate logistic regression for DN in T2DM patients.

**Variable**	**B**	**SE**	**Wald**	**P**	**OR**	**Lower**	**Upper**
Age (>60)	0.313	0.189	2.738	0.098	1.37	0.94	1.98
Gender (Male)	0.401	0.190	4.478	0.034	1.49	1.03	2.17
Smoking (Yes)	0.903	0.193	21.852	<0.001	2.47	1.69	3.60
Drinking (Yes)	-0.089	0.192	0.215	0.643	0.92	0.63	1.33
BMI (>28)	0.607	0.212	8.191	0.004	1.84	1.21	2.78
SBP (>140)	0.803	0.186	18.585	<0.001	2.23	1.55	3.22
DBP (>90)	0.439	0.215	4.149	0.042	1.55	1.02	2.37
FBG (>6.9)	0.485	0.195	6.175	0.013	1.62	1.11	2.38
HbA1c (6.9%)	0.218	0.183	1.415	0.234	1.24	0.87	1.78
TC (>4.5)	0.986	0.192	26.264	<0.001	2.68	1.84	3.91
TG (≥1.7)	0.768	0.191	16.117	<0.001	2.16	1.48	3.14
HDL (≥1.0)	1.422	0.743	3.662	0.056	4.14	0.97	17.78
LDL≥ (1.8)	-0.041	0.193	0.046	0.831	0.96	0.66	1.40
BUN (>7.1)	0.897	0.220	16.686	<0.001	2.45	1.60	3.77
SCR (>106)	0.286	0.336	0.723	0.395	1.33	0.69	2.57
UC (>440)	0.060	0.159	0.142	0.707	1.06	0.78	1.45
Risk Score	0.999	0.093	115.102	<0.001	2.72	2.26	3.26

### Assessment of clinical application of prediction model

**Figure 6:**
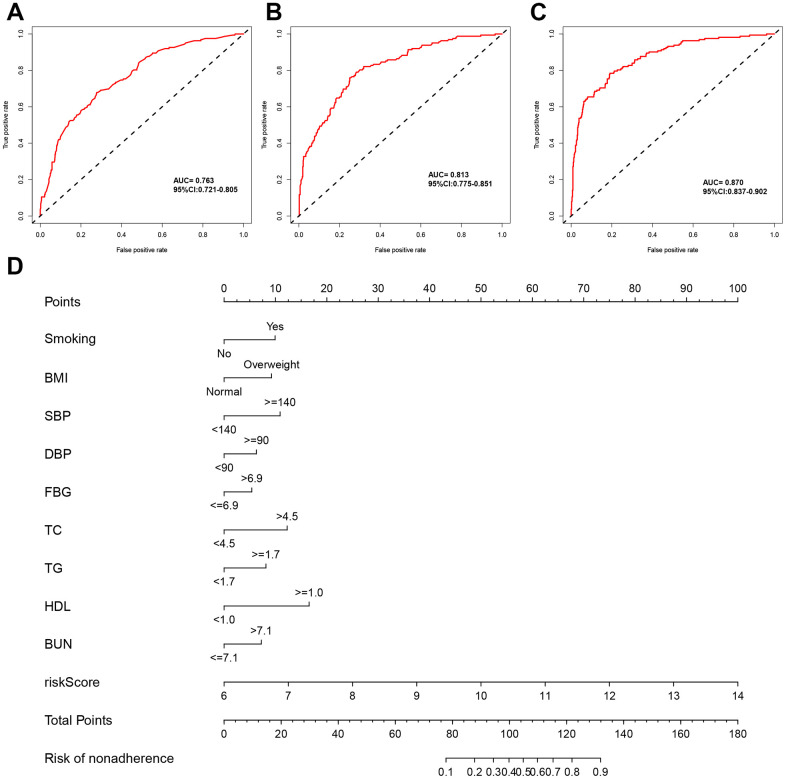
**Development of DN risk model and nomogram prediction based on serum mRNA of hub genes.** ROC curves of DN risk models in T2DM based on (**A**) clinical parameters, (**B**) risk score of hub genes, and (**C**) clinical parameters and risk score of hug gens. (**D**) Nomogram of DN risk model in T2DM.

To validate the model, we built the following three models: model 1: risk score, mode 2: clinical parameters; model 3: risk score and clinical parameters. For models 1 and 2, the AUCs were 0.763 (95%CI: 0.721-0.805, Figure 6A) and 0.813 (95%CI: 0.775-0.851, Figure 6B) respectively. Model 3 showed the highest predicting ability (AUC:0.870, 9%CI: 0.837-0.902, Figure 6C). Based on model 3, we built a nomogram plot to evaluate the risk probability of DN for individuals (Figure 6D). We only included the significant variables in the univariate logistic regression into the nomogram. Specifically, we can get a point for each variable. We summarized the points of all variables in the nomogram. The summarized points can be projected onto the risk of nonadherence. Then we can know the risk probability of individuals. These descriptions were added in the manuscript.

**Figure 7:**
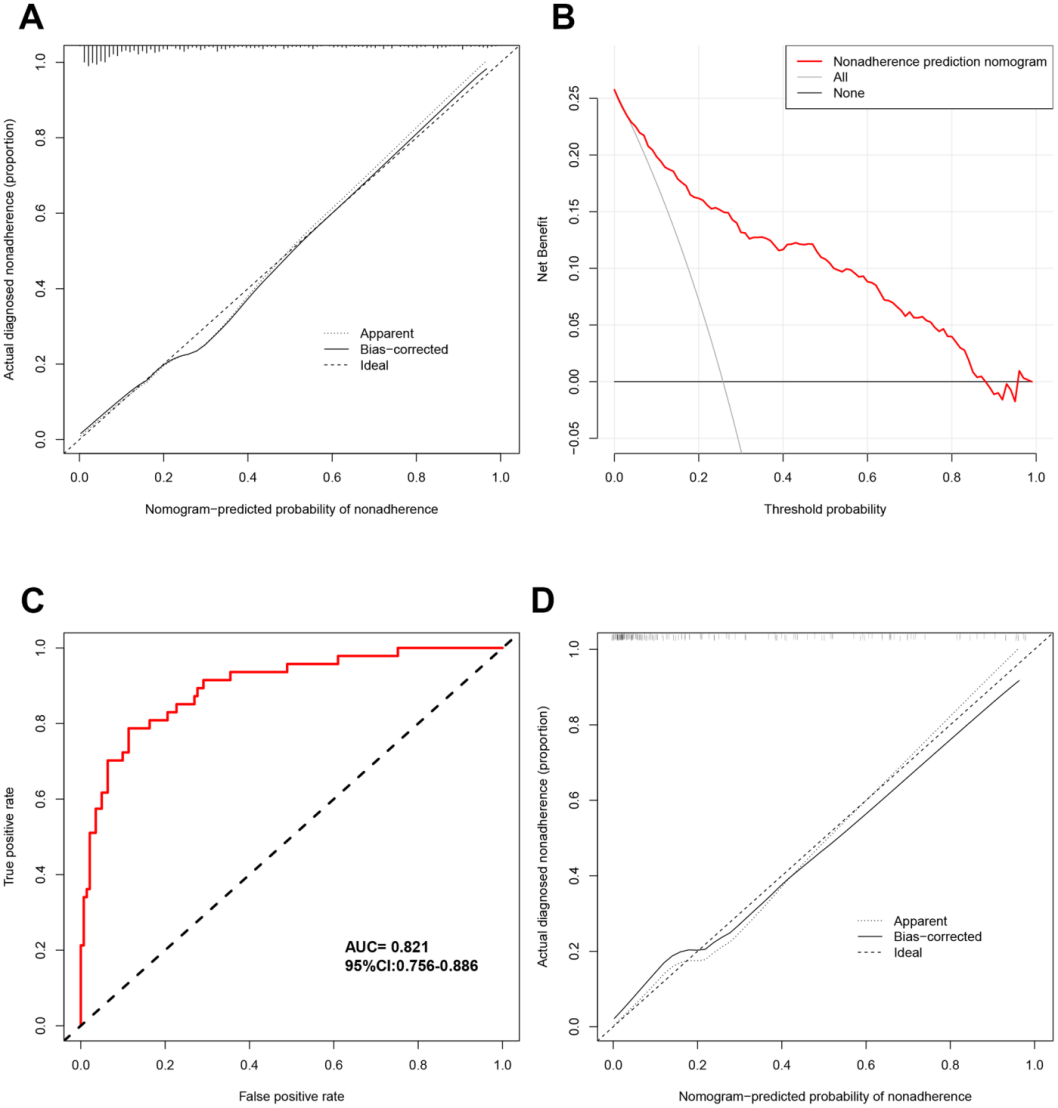
**Assessment and validation of DN risk model.** (**A**) Calibration curves of DN risk prediction in the whole cohort. (**B**) Decision curve analysis for DN risk model in whole cohort. (**C**) ROC curves of DN risk model in validated cohort. (**D**) Calibration curves of DN risk prediction model in the validated cohort.

Furthermore, we evaluated the agreement between actual diagnosed probability and nomogram-predicted probability. Results showed relatively high agreement (Figure 7A). The calculated C-index was 0.870, suggesting that the model has high prediction accuracy. Moreover, decision curve analysis for DN risk indicated that T2DM patients will benefit from this DN risk prediction model when the threshold probability of a patient and clinician was 25%, meaning the net benefit is relatively high based on the DN risk nomogram (Figure 7B). To further assess the model, we randomly extracted half of the whole study sample to validate the prediction model. The internal validation showed that the AUC was 0.821 (95%CI: 0.756-0.886, Figure 7C), and the calibration curve of this sample also indicated high performance (Figure 7D). These results suggest that the DN prediction model can help clinicians make decision for T2DM patients.

## DISCUSSION

According to the International Diabetes Federation, the global prevalence of diabetes is expected to rise to 10.2% (about 578 million people) by 2030 and to 10.9% (about 700 million people) by 2045 [17]. At present, the prevalence of diabetes in China is up to 11.2%. The awareness rate, control rate and treatment rate of diabetes have improved, but are still low [18]. About 30% of diabetic patients can develop DN, which is closely related to the high incidence and mortality of heart disease, and then develop end-stage renal disease. Our results show the DN incidence of T2DM patients is 25.7%, which falls into the scope. Therefore, early detection and screening of high-risk patients with DN is the key to prevention and treatment.

DN is the main cause of end-stage renal disease. In the early stage of DN, renal function damage can be alleviated or even reversed. Once at the stage of clinical macroalbuminuria, renal function damage will be progressively aggravated and become irreversible in the environment of continuous hyperglycemia [19]. Thus, it is of great important of establish an evaluation tool for predicting early-stage DN. We explored the differences of serum transcriptomics between DN and non-DN in T2DM patients, and obtained 110 target genes using WGCNA. GO enrichment analysis indicates that these target genes are mainly related to membrane adhesion, alpha-amino acid biosynthesis, metabolism, and binding, terminus, inhibitory synapse, clathrinid-sculpted vesicle, kinase activity, hormone binding, receptor activity, and transporter activity. KEGG analysis indicates synaptic vesicle cycle, cysteine and methionine metabolism, N-Glycan biosynthesis, osteoclast differentiation, and cAMP signaling pathway can be involved in the process of DN in diabetic patients. Next, we detected the expression levels of hub genes in a retrospective cohort, and developed an early DN prediction model for T2DM patients. Finally, we found the prediction model can be well applied in clinical practice, as confirmed by internal validation and several other methods.

A prospective investigation performed in 28 countries indicates that the prevalence of DN in T2DM is 27.9%. The prevalence differs among countries or areas. The prevalence of DN is the highest in Russia and Middle East, but is the lowest in South Asia [20]. The data from USA show that the prevalence of DN is 23.7% among diabetic patients [21]. In South Korea, the prevalence of DN is 26.7% in diabetic patients. Our study shows that the prevalence of DN is 25.8%, which is close to the level of South Korea [22]. However, the prevalence is 20% in European countries, which ranks the first in all complications of diabetes. These results indicate the prevalence of DN varies among different populations.

Nomograms have been extensively used in the prognosis prediction of tumor and non-tumor diseases. Nomograms can well express the relationships between variables and clinical outcomes [23]. There are already some DN prediction models in T2DM patients. Jiang S et al. enrolled 302 T2DM patients who underwent continuous renal biopsy in China-Japan Friendship Hospital, and randomly divided their data into a training set with 70% of patients and a validation set with the remaining 30% for model construction and external validation. Nine variables including gender, course of diabetes, diabetic retinopathy (DR), hematuria, HbA1c, hemoglobin, blood pressure, urinary protein excretion and eGFR were included to construct a Nomogram. The C index of the model was 0.934, and the C index of internal and external verification was 0.91 and 0.875 respectively [24]. Xi C et al. conducted a questionnaire including physical examination, blood routine examination and biochemical index evaluation of 1095 T2DM patients in Guilin. Risk factors such as gender, age, hypertension, duration of drug use, diabetes, BMI, blood urea nitrogen, serum creatinine levels, the ratio of neutrophils and lymphocytes and red blood cell distribution width were combined with selected risk factors and involved into the logistic regression analysis of a prediction nomogram model. The C index was 0.819. The area under the ROC curve (AUC) was 0.813, and the C index of internal verification was 0.796. Decision curve analysis showed the risk profile of DN was clinically appropriate when the risk threshold ranged from 1% to 83% [25]. These two studies show that nomograms play a certain role in the risk assessment of DN. However, the sample sizes of these two studies are small, and the second study has no external verification. Certainly, there are community-based studies with large sample sizes. Shi R et al. included 4219 T2DM patients, and divided them into a T2DM group, a DK group, a DR group and a DR+DN group. The predictive factors in the prediction model included course of disease, BMI, triglyceride, systolic blood pressure, postprandial blood glucose, HbA1c and urea nitrogen. The C index of the model is 0.807, the THE AUC is 0.807, the C index of internal validation is 0.804, and the analytical risk threshold of the decision curve is 16%-75%, indicating this model can predict DK risk [26]. In addition, Wang G et al. selected 2163 diabetic inpatients and established 4 screening equations based on Nomograms. The factors included in the four models were different, and 10 factors were included in the whole model: drinking status, hypertension, duration of diabetes, history of coronary heart disease, SBP, total cholesterol, fasting plasma C-peptide, uric acid, and diabetic retinopathy. Model 1 included 7 factors: sex, SBP, TC, alcohol consumption, hypertension, coronary heart disease and duration of diabetes. HbA1c was added to model 2 based on Model 1. There were 6 factors in the simplified model: sex, SBP, alcohol consumption, hypertension, coronary heart disease and duration of diabetes. The C index of the four models is 0.8450, 0.8149, 0.8171 and 0.8083, respectively, which are 3.2756, 7.749, 10.023 and 12.294 according to Hosmer-Lemeshow goodness of fit test [27]. Both model 1 and Model 2 have good predictive performance and validity, and can be used to screen DKD cases in China. Despite the large sample sizes, these two studies were not verified externally, so their applicability needs further consideration.

We built a risk score for DN in T2DM patients using six genes. The univariate and multivariate logistic regressions showed that risk score was an independent risk factor of DN. To develop a risk prediction tool of DN, we built three models: model 1 based on clinical characteristics; model 2 based on hub genes, and model 3 based on both clinical characteristics and hub genes. The values of the C-index are 0.781, 0.815, and 0.872, respectively. The AUCs are 0.763, 0.813 and 0.870. Like previous studies, our multivariate regression indicates similar risk factors, such as smoking status, overweight, elevated SBP, TC, TG, and BUN [28–30]. There are several differences between our study and previous studies. Our study includes clinical characteristics and gene expression. Our data of gene expression were obtained from the serum detection, which is easily achieved compared with tissue biopsy. Our study also has several limitations: the model validation is based on interval data, but external validation is required. Some factors included the model may be inapplicable into community research.

In conclusion, we established a novel DN risk model with relatively high prediction accuracy based on clinical characteristics and hub genes of serum detection. The estimated risk score can help clinicians develop individualized intervention programs for DN in T2DM. External validation data are required before individualized intervention measures are taken.
